# How Is Sleep Related to Anxiety or Depression in Older Adults With Dementia?

**DOI:** 10.1093/geroni/igaa057.2106

**Published:** 2020-12-16

**Authors:** Yeji Hwang, Nancy Hodgson

**Affiliations:** 1 University of Pennsylvania, Philadelphia, Pennsylvania, United States; 2 University of Pennsylvania, Cherry Hill, New Jersey, United States

## Abstract

Anxiety and depression are one of the most distressing symptoms for the family caregivers. Little is known about the relationship between sleep impairments and anxiety/depression in this population and how objective and subjective sleep measures differ in relation to anxiety. This study was designed to examine the relationship between sleep impairments and anxiety/depression in people with dementia, using both subjective and objective sleep measures. Among the 170 study participants, 50% (n=85) reported to have anxiety/depression. In univariate logistic regression analyses on anxiety/depression, adjusting for dementia stage, people with more subjective sleep impairment had higher odds of having anxiety/depression (OR=1.111; 95% CI: 1.020-1.211, p=0.016) and people with poorer subjective sleep quality had higher odds of having anxiety/depression (OR=1.702; 95% CI: 1.046-2.769, p=0.032). Objective sleep measures from actigraphy did not show any significant relationships to anxiety/depression. The results suggest that subjective sleep measures are closely related to anxiety/depression in this population.

